# Topical Application of a Vitamin A Derivative and Its Combination With Non-ablative Fractional Laser Potentiates Cutaneous Influenza Vaccination

**DOI:** 10.3389/fmicb.2018.02570

**Published:** 2018-10-30

**Authors:** Peiyu Li, Ji Wang, Miao Cao, Qiwen Deng, Shibo Jiang, Mei X. Wu, Lu Lu

**Affiliations:** ^1^Key Laboratory of Medical Molecular Virology of MOE/MOH, School of Basic Medical Sciences & Shanghai Public Health Clinical Center, Fudan University, Shanghai, China; ^2^Wellman Center for Photomedicine, Massachusetts General Hospital, Department of Dermatology, Harvard Medical School, Boston, MA, United States; ^3^Department of Infectious Diseases and the Key Lab of Endogenous Infection, Shenzhen Nanshan People’s Hospital, Guangdong Medical University, Shenzhen, China; ^4^The First Affiliated Hospital of Sun Yat-sen University, Sun Yat-sen University, Guangzhou, China; ^5^Lindsley F. Kimball Research Institute, New York Blood Center, New York, NY, United States; ^6^Harvard-MIT Division of Health Sciences and Technology, Cambridge, MA, United States

**Keywords:** all-*trans* retinoic acid, cutaneous adjuvant, influenza vaccine, interferon regulatory factor 3, interferon regulatory factor 7, non-ablative fractional laser, type I interferon

## Abstract

Skin contains a large number of antigen presenting cells, making intradermal (ID) injection one of the most effective ways for vaccine administration. However, although current adjuvants may cause severe local reactions and inflammations in the skin, no adjuvant has been approved for ID vaccination so far. Here, we report that topical application of all-*trans* retinoic acid (ATRA), a vitamin A derivative produced in the human body, augmented cutaneous influenza vaccination. The adjuvant effects were evaluated in a murine vaccination/challenge model by using A/California/07/2009 pandemic vaccine (09V) or a seasonal influenza vaccine (SIV). ATRA drove a Th2-biased immune response, as demonstrated by profoundly elevated IgG1 titer rather than IgG2 titer. Combining ATRA with a non-ablative fractional laser (NAFL), which represents a new category of vaccine adjuvant utilizing physical stimuli to induce self-immune stimulators, further enhanced the efficacy of influenza vaccines with a more balanced Th1/Th2 immune response. The dual adjuvant strengthened cross-reactive immune responses against both homogenous and heterogeneous influenza viral strains. Analysis of gene expression profile showed that ATRA/NAFL stimulated upregulation of cytosolic nucleic acid sensors and their downstream factors, leading to a synergistic elevation of type I interferon expression. Consistent with this finding, knocking out IRF3 or IRF7, two key downstream regulatory factors in most nucleic acid sensing pathways, resulted in a significant decrease in the adjuvant effect of ATRA/NAFL. Thus, our study demonstrates that the self molecule ATRA could boost cutaneous influenza vaccination either alone or ideally in combination with NAFL.

## Introduction

Vaccination is the most effective way to prevent pathogen infections, such as influenza viruses which can cause worldwide circulation and infection with an annual infection rate estimated at 5–10% in adults and 20–30% in children^1^, resulting in severe morbidity and mortality (Iuliano et al., 2018). Owing to their expansive genetic diversity, rapid antigenic drift and shift across subtypes, influenza viruses are prone to generate new subtypes continuously, a phenomenon which poses a substantial biological threat to public health (Lu et al., 2012; Uyeki et al., 2017; Petrova and Russell, 2018). Wider vaccination has been suggested to mitigate the burden of influenza-related morbidity and mortality (Monto et al., 2009; Pawelec and McElhaney, 2018; Sullivan, 2018); therefore, continuous updating is required to keep pace with the evolution of the circulating and mutated viruses (Paules et al., 2017; Xu et al., 2017). Moreover, many existing vaccines initiate weak immune responses that do not offer adequate protection against viral infections. This requires the use of safer and more effective adjuvants (McKee et al., 2007; Reed et al., 2013; He et al., 2016), especially for those influenza vaccines with low immunogenicity (Petrovsky and Aguilar, 2004; Di Pasquale et al., 2015). Most current vaccines are administered via intramuscular injection (IM). In fact, skin comprises a network of immune cells, and such network plays an important role in host defense against pathogenic invaders (Pasparakis et al., 2014). Vaccines delivered by intradermal injection (ID) are more effective than those administered by intramuscular injection (IM) based on minimizing the doses of vaccine used (dose-sparing), the significance of which is notable in the case of unanticipated vaccine shortages during influenza pandemics (Kenney et al., 2004; Hung et al., 2012; Williams, 2013). However, most adjuvants are not compatible with ID immunization strategies because they are prone to cause severe local reactions at the injection site, including erythema, swelling, and even ulcers for several weeks (Andrianov et al., 2009; Wang et al., 2016b). To date, no adjuvant has been approved for cutaneous vaccination, calling for the development of new and safe adjuvants to accompany a growing number of vaccines using novel intradermal immunization technologies like microneedle arrays (McAllister et al., 2014; Galvez-Cancino et al., 2018; Golombek et al., 2018).

All-*trans* retinoic acid (ATRA), also known as retinoic acid (RA) or vitamin A acid, is a ligand for both the retinoic acid receptor (RAR) and the retinoid X receptor (RXR), and it is the active metabolic intermediate of vitamin A in animals (Hall et al., 2011). Vitamin A and its metabolite RA are known to play important roles in the mucosal immune system, mainly in regulating T-cell homing (Johansson-Lindbom and Agace, 2004; Svensson et al., 2008; Zeng et al., 2013), priming T cell migration into the epidermis (Sigmundsdottir and Butcher, 2008) and regulating intestinal and skin dendritic cells (Raverdeau and Mills, 2014; Bakdash et al., 2015; Zeng et al., 2016). Moreover, vitamin A was shown to synergize with catechin as a vaccine adjuvant to enhance immunity (Patel et al., 2016), and RA also has an adjuvant role in a broad spectrum of biological functions, including induction of Th1 and Th2 effector T cells (Erkelens and Mebius, 2017). Additionally, vitamin A has been recognized as an essential nutrient for immune responses for nearly 100 years (Green and Mellanby, 1928; Hashimoto-Hill et al., 2017), and ATRA (Tretinoin), a medication used for the treatment of acne and photodamaged wound of skin (Cho et al., 2005), has been approved by the U.S. Food and Drug Administration (FDA) for application to the skin in the form of a cream^2^. It is easy to obtain and has been proven to be extremely safe after years of validation and application. Moreover, the RA inducible gene I (RIG- I), which can be activated and induced by ATRA, plays an important role in innate immune defense against viral infections as a sensor of viral nucleic acids (Errett and Gale, 2015). These evidences suggest that ATRA has the potential to be used as a promising cutaneous adjuvant and its adjuvant efficiency should be determined.

The laser has been recently developed as a new category of vaccine adjuvant that activates the immune system without introducing non-self molecules into human body (Chen et al., 2010, 2012, 2013; Wang et al., 2014, 2015, 2016a,b; Kashiwagi et al., 2016; Kim et al., 2016; Lopes et al., 2018). Of these vaccine adjuvants, the non-ablative fractional laser (NAFL) is very promising. The micro-sterile inflammation-induced array of self-renewable microthermal zones (MTZs) generated by NAFL will attract a large number of antigen-presenting cells at the vaccination site (Wang et al., 2014). Meanwhile, dead cells in MTZs release nucleic acid, such as dsDNA, and activated nucleic acid sensing pathways, such as the cGAS/STING pathway, to induce type I interferons (Roers et al., 2016). Although the use of NAFL adjuvant alone has significant advantages owing to its super safety, efficacy can still be improved. Therefore, it is necessary to explore a natural biological or chemical-free substance that can enhance its adjuvanticity. ATRA treatment is well known to induce many genes involving nucleic acid sensing which is the major mechanism underlying the adjuvant effect of NAFL, suggesting a possible synergy between NAFL and ATRA.

Given the safety and immune-stimulatory effects, we here evaluate the potential of topical ATRA and its combination with NAFL as a safe and effective adjuvant for cutaneous influenza vaccination. The murine vaccination/challenge model is enrolled to demonstrate the adjuvant effect of ATRA/NAFL for both pandemic and seasonal influenza vaccines (SIVs). Furthermore, the underlying molecular mechanism of ATRA/NAFL is explored by an extensive screening of related pathways. The ATRA/NAFL is demonstrated to be safe and effective with a clear mechanism involving nucleic acid-sensing pathways and type I interferons.

## Materials and Methods

### Animals

Inbred C57BL/6 mice and outbred Swiss Webster female mice at 6–8 weeks of age were purchased from Charles River or Jackson Laboratories. IRF3^-/-^ and IRF7^-/-^ mice on the C57BL/6J background were a kind gift of Dr. T. Taniguchi, Tokyo University (Honda et al., 2005). All mice were maintained under specific pathogen-free conditions at the animal facilities of Fudan University and Massachusetts General Hospital (MGH). All studies were approved and monitored by Fudan University and MGH Institutional Animal Care and Use Committee (IACUC).

### Influenza Vaccines and Viruses

A/California/7/2009 H1N1, A/New Caledonia/20/1999 H1N1, and A/Puerto Rico/8/1934 H1N1 influenza viruses were obtained from American Type Culture Collection (ATCC) and BEI Resources. They were propagated in 10-day-old embryonated chicken eggs (Charles River Laboratories, Boston; Beijing Merial Vital Laboratory Animal Technology Co., Ltd.) at 35°C for 3 days, harvested, purified by sucrose gradient ultracentrifugation, and stored at -80°C until use. A/California/7/2009 H1N1 influenza vaccine was currently obtained from influenza viruses collected as described above after inactivation with 0.025% formalin and dialysis with PBS. Seasonal influenza vaccine (2011–2012 formulation) was purchased from GlaxoSmithKline (Research Triangle Park, NC), containing three strains, A/California/07/2009, A/Victoria/210/2009, and B/Brisbane/60/2008.

### Laser Device and Adjuvants

The powder and cream of ATRA were purchased from Sigma (R2625) and Perrigo (NDC 45802-361-02), respectively. An FDA-approved, home-use, hand-held NAFL (PaloVia Skin Renewing Laser, Palomar Medical Technologies) was used as adjuvant via skin in mice. The device emits laser light in the wavelength of 1410 nm, and 2 hits at high power were used at the inoculation site in the skin before vaccination to create an array of self-healing microthermal zones (MTZs).

### Mice Immunization and Challenge Procedures

6–8-week-old C57BL/6, Swiss Webster, IRF3^-/-^ and IRF7^-/-^ knockout mice were anesthetized intraperitoneally (IP) with 100 mg/kg ketamine and 10 mg/kg xylazine. The hair on the back of mice was removed and illuminated with NAFL on the lower back of the skin on the next day before the vaccine was ID immunized with a 20 μl dose, including 300 ng A/California/7/2009 H1N1 vaccine or 5 μl SIV at 6–8 weeks. After ID immunization, the inoculation site was either left alone or topically applied with ATRA cream (Perrigo), and then the site was covered with Tegaderm film (3M) for 4 h. Mice were challenged with a dose of 20 μl 10 × LD50 A/California/7/2009 H1N1 virus 2 weeks post-immunization of A/California/7/2009 H1N1 vaccine, and the survival rate was monitored for 2 weeks.

### Blood Collection and ELISA Assays

Blood was collected by retro-orbital sinus 2 weeks post-immunization, and serum was separated by centrifuge after clotting for 1–2 h at room temperature. Influenza virus-specific antibodies were determined by ELISA. Briefly, A/California/7/2009 H1N1 vaccine or influenza seasonal vaccine was coated on 96-well plates and blocked by 5% skimmed milk. Serum was diluted by 1:100 and then diluted by fourfold, incubated for 2 h at room temperature. After washing, anti-mouse IgG (NA931V, GE Healthcare, dilution 1:6000), IgG1 (1073-05, Southern Biotech, 1:4000), IgG2a (61-0220, Life Technologies, dilution 1:2000), IgG2c (1079-05, Southern Biotech, 1:5000) were added with HRP-conjugated Streptavidin into plates for 1 h. One SIGMAFAST^TM^ OPD (o-Phenylenediamine dihydrochloride) tablet and one urea hydrogen peroxide/buffer tablet (P9187-50set, Sigma) were dissolved in 20 ml of water to prepare the reaction buffer, and 50 μl buffer was added into each well for reaction. Fifteen minutes later, the reaction was stopped by 50 μl 2M H_2_SO_4_. The optical density is A490.

### Hemagglutination Inhibition (HAI) Assay

Hemagglutination is the process by which influenza virus (and other viruses) causes red blood cells to agglutinate. In short, serum samples were treated with receptor-destroying enzyme (RDE) (Denka Seiken) for 18–24 h at 37°C, followed by treatment at 56°C for 30 min to remove complement and inactivate RDE. The resultant serum samples were serially diluted and incubated with 4 hemagglutination units (HAU) of influenza virus A/California/7/2009 H1N1, A/New Caledonia/20/1999 H1N1 or A/Puerto Rico/8/34 H1N1 for 30 min at room temperature. Then the mixture was incubated with 0.5% chicken red blood cells (Lampire Biological Laboratories) at room temperature for 30 min. The HAI titer was defined as the reciprocal of the highest serum dilution that inhibited 4 HAU.

### Histological Examination

Mice were treated with ATRA, NAFL, ATRA/NAFL for 24 h. The inoculation sites were photographed after 24 h treatment. The skin at the inoculation site was dissected 24 h after treatment, fixed, and stained by a standard H&E procedure.

### mRNA Expression Level at Skin Inoculation Site

The lower dorsal skin of C57BL/6 mice was treated by ATRA adjuvant, NAFL adjuvant, and ATRA/NAFL adjuvant, respectively. Twenty-four hours later, the skin at the inoculation site was isolated, excised, and total RNA was extracted by the RNeasy Mini Kit (Qiagen). The cDNA was synthesized by using the High Capacity RNA-to-cDNA Kit (Applied Biosystems). The mRNA expression level was amplified and determined by real-time PCR using an SYBR Green PCR Kit (Roche). Glyceraldehyde 3-phosphate dehydrogenase (GAPDH) was used as an internal control. A heat map diagram of the genes of interest was illustrated with HemI software. See Table 1 for primer sequences of all genes.

**Table 1:** Primers for qPCR analysis.

Gene	Forward primer	Reverse primer
***Aim2***	ACGTTGTTAAGAGAGCCAGGG	AGCACCAACACCTCCATTGT
***Cgas***	GGAAGCCCTGCTGTAACACT	CCAGCCAGCCTTGAATAGGT
***Cxcl-10***	CCAAGTGCTGCCGTCATTTTC	TCCCTATGGCCCTCATTCTCA
***Ddx41***	CTCTCGCTTGGAGAAAGGCA	CCTACCATTCTGTCCGGTCG
***Dhx9***	TTGAAATTGTGCCCCCACCT	GTGACCAAGGAACCACTCCC
***Gapdh***	*ATCAAGAAGGTGGTGAAGCA*	AGACAACCTGGTCCTCAGTGT
***Ifnb1***	CAGCTCCAAGAAAGGACGAAC	GGCAGTGTAACTCTTCTGCAT
***Irf1***	CCAGCTCTTGCTTTCGGACG	TCGAGTGATTGGCATGGTGG
***Irf3***	AACCGGAAAGAAGTGTTGCG	CCCTGGAGTCACAAACTCATAC
***Irf7***	TCCAGTTGATCCGCATAAGGT	CTTCCCTATTTTCCGTGGCTG
***Ku70***	CACCAAGCGGTCTCTGACTT	AGAGAGGGCCTCAGGTAGTG
***Mavs***	CTGCCTCACAGCTAGTGACC	CCGGCGCTGGAGATTATTG
***Mre11***	CGGTCAATGTCGGTGGAGAA	CCTAAGCCGTACAGAGCGAG
***Myd88***	AGGACAAACGCCGGAACTTTT	GCCGATAGTCTGTCTGTTCTAT
***Pol III***	TGGCTTGCGACTTGGAGAAA	GTAGTGGCACCAGCCAGAAT
***Rig-I***	CCACCTACATCCTCAGCTACATGA	TGGGCCCTTGTTGTTCTTCT
***Sting***	TCTCCTGTCTAACCCCTCCC	GGATTTCCAGAGGCCCCAAA
***Tbk1***	*GTACGGCACAGAAGAGTACCT*	ATGGTAGAATGTCACTCCAACAC
***Tnf-α***	CCTGTAGCCCACGTCGTAG	GGGAGTAGACAAGGTACAACCC
***Traf3***	CGTGCCGACTGCAAAGAAAA	TTGATCATGGGCACTTGGCT
***Zbp1***	TACCGCCTGAAGAAGGAGGA	TTCTCAGGGATTGCAGGAGC

### Statistical Analysis

A two-tailed *t*-test (*t*-test) was used to analyze the difference between two groups. One-way ANOVA was used among multiple groups involving one factor, and two-way ANOVA was used to analyze experiments involving two factors each with multiple groups. Percentages of survival were analyzed by log-rank test. *P*-value was calculated by PRISM software (GraphPad), and the difference was considered significant if the *p*-value was less than 0.05.

## Results

### The Adjuvant Effect of ATRA/NAFL for Pandemic Influenza Vaccine

**FIGURE 1:**
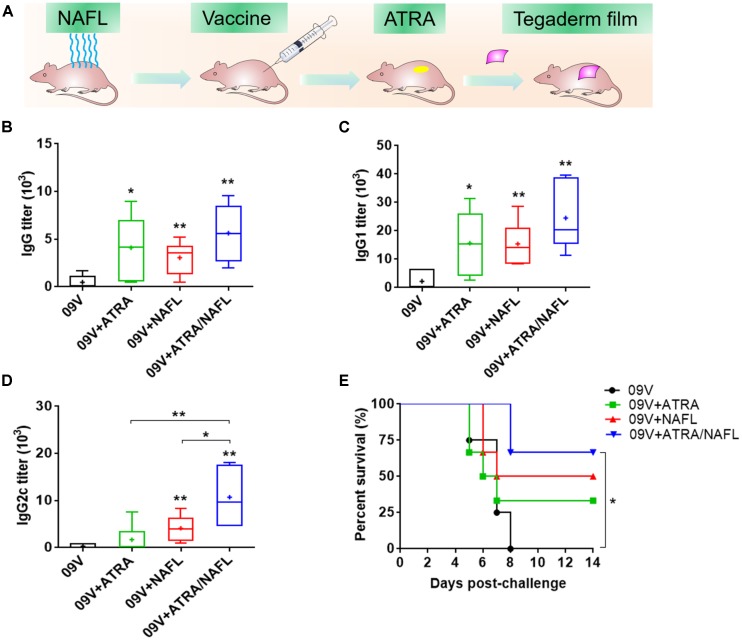
NAFL and ATRA enhanced protective immunity on pandemic 2009 H1N1 vaccine in C57BL/6 mice. 300 ng A/California/7/2009 H1N1 vaccine (HA content) was immunized i.d. alone or with adjuvant ATRA, NAFL (2 hits, high) or ATRA/NAFL (2 hits, high) as illustrated in **(A)**, and then vaccine-specific antibody titer for **(B)** IgG, **(C)** IgG1 and **(D)** IgG2c was determined 2 weeks post-immunization. Data are presented as box and whiskers plots, in which whiskers are min-to-max. Three lines of the box are the 25th percentile, the median, and the 75th percentile, respectively. Mean is shown as “ + ”. The immunized mice alone or with different adjuvants were intranasally challenged with 10 × LD50 of A/California/7/2009 H1N1 virus 2 weeks post- immunization, and **(E)** survival rate of the challenged mice was monitored every day for 2 weeks. *N* = 6. ^∗^*P* < 0.05 or ^∗∗^*P* < 0.01, respectively.

To determine the individual effect of ATRA and NAFL, respectively, and the synergistic effect of ATRA/NAFL in augmenting the efficacy of influenza vaccines, C57BL/6 mice were first treated with or without NAFL following by injection of A/California/7/2009 H1N1 pandemic influenza vaccine and topical application of ATRA ointment or PBS at the inoculation site (Figure 1A). As shown in the figures (Figure 1B–D), influenza vaccine with ATRA alone significantly elevated the vaccine-specific IgG and IgG1 titers, which is roughly equal to the titers lifted by NAFL alone, but not IgG2c, suggesting that ATRA chiefly induces Th2-dominated immune responses. NAFL significantly enhanced the efficacy of 2009 vaccine with both influenza-specific IgG1 and IgG2c antibody titers elevated (Figure 1C,D), consistent with our previous report (Wang et al., 2014). Although ATRA or NAFL alone can promote vaccine immunization, the promotion needs to be further strengthened. Surprisingly, ATRA/NAFL combination augmented influenza-specific IgG, IgG1, and IgG2c antibody titers. Particularly, ATRA/NAFL combination significantly enhanced the higher IgG2c antibody titers than either ATRA or NAFL alone, being considered as promoting Th1 immune response (Figure 1D).

Next, the immunized mice were challenged by a mouse-adapted A/California/7/2009 H1N1 viruses 2 weeks post-vaccination. The survival curve (Figure 1E) displayed that all mice in 2009 vaccine alone group died. While vaccination with ATRA or NAFL alone slightly elevated the survival rate, only ATRA/NAFL combination achieved significant protection (Figure 1E). These results proved that the combination of ATRA and NAFL synergistically enhanced immune responses induced by the pandemic influenza vaccine.

### The Safety of ATRA/NAFL in Mice

**FIGURE 2:**
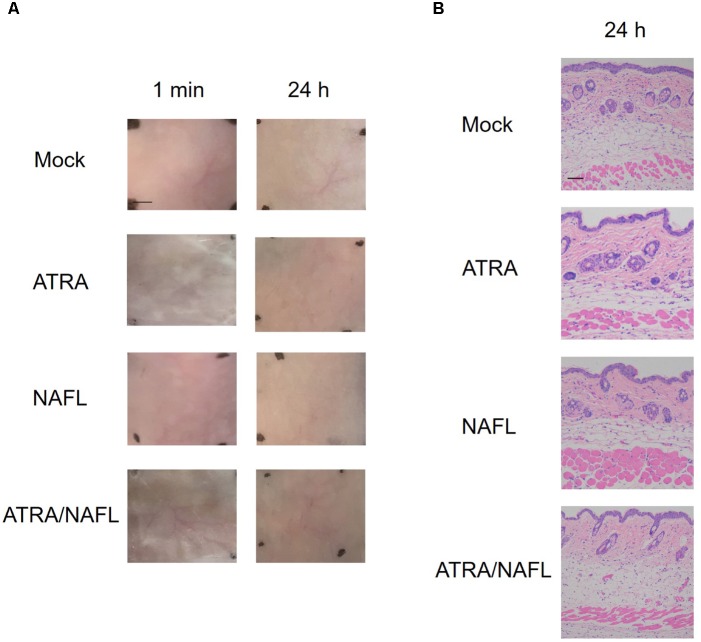
Histology examination of NAFL/ATRA treated skin. Mice were treated with ATRA, NAFL, ATRA/NAFL, respectively. Then the ATRA-treated site was covered with Tegaderm film (3 M) for 4 h. **(A)** Photos were taken at indicated time points, and representative photos are shown with four mice in each group. Scale bar, 0.5 cm. **(B)** Representative hematoxylin and eosin-stained sections of the inoculation site 24 h post-treatment. Scale bar, 200 μm.

The safety of ATRA/NAFL in cutaneous vaccination was next evaluated. ATRA, NAFL, ATRA/NAFL were applied onto the skin for 24 h. As shown in Figure 2A, NAFL treatment only induced slight skin swelling which quickly dissipated in 10 min without causing any erythema or other obverse local reactions. On the other hand, ATRA treatment did not induce any observable skin irritations either. Encouragingly, the combination of ATRA and NAFL was comparable with NAFL treatment alone in terms of local reactogenicity. The safety of the combination was further confirmed by histological examination, in which neither infiltration of inflammatory cells nor morphology change of epidermis and dermis was found in the skin 24 h after adjuvants treatment (Figure 2B).

### The Adjuvant Effect of ATRA/NAFL for Seasonal Influenza Vaccine

**FIGURE 3:**
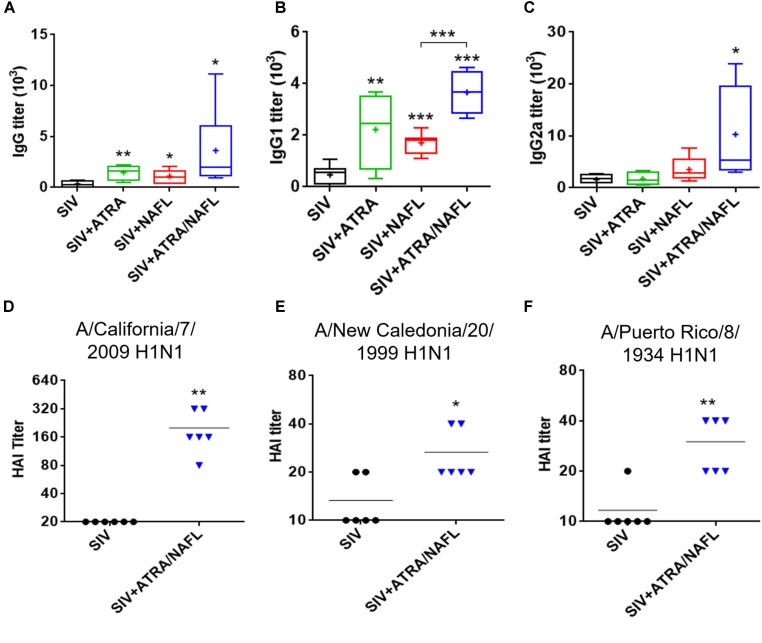
ATRA and NAFL enhanced immune responses of seasonal influenza vaccine (SIV). 5 μl SIV was immunized i.d. alone or with adjuvant ATRA, NAFL (2 hits, high) or ATRA/NAFL (2 hits, high) in 6- to 8-week-old female Swiss Webster (SW) mice, and vaccine-specific antibody titer for **(A)** IgG, **(B)** IgG1 and **(C)** IgG2a was determined 2 weeks post-immunization. *N* = 7, except for the SIV + ATRA group (*N* = 4). Data are presented as box and whiskers plots, in which whiskers are min-to-max. Three lines of the box are the 25th percentile, the median, and the 75th percentile, respectively. Mean is shown as “ + ”. HAI titers of sera were determined by HAI assays against **(D)** A/California/7/2009 H1N1, **(E)** A/New Caledonia/20/1999 H1N1 and **(F)** A/Puerto Rico/8/1934 H1N1 influenza viruses 2 weeks post-immunization. Data are represented as mean. *N* = 6. Each symbol represents data from individual mice, and horizontal bars indicate mean. ^∗^*P* < 0.05, ^∗∗^*P* < 0.01 or ^∗∗∗^*P* < 0.001, respectively.

Next, the adjuvant effect of ATRA and ATRA/NAFL was also explored in SIVs. As shown in Figure 3A, although both ATRA and NAFL slightly increased IgG titers by twofold, only the ATRA/NAFL combination showed a significant adjuvant effect and elevated IgG titers by tenfold. Consistent with the result of pandemic influenza vaccine, ATRA mainly enhanced Th2 immune responses, but not Th1, as indicated by elevated IgG1 titers (Figure 3B,C). On the other hand, the ATRA/NAFL combination greatly enhanced both Th1 and Th2 immune responses by sixfold or eightfold, respectively, compared to vaccine alone (Figure 3B,C), but also significantly enhanced the higher IgG1 titer over NAFL adjuvant (Figure 3B). Sera were next measured for hemagglutination inhibition (HAI) titer, a standard criterion of influenza vaccination, in which a serum HAI titer greater than, or equal to, 1:40 is considered protective. The protective immune responses were subsequently assessed by measuring HAI titer against both homogeneous and heterogeneous H1N1 strains. Strikingly, ATRA/NAFL greatly elevated HAI titers in an immunization condition (Figure 3D). Moreover, antibodies induced by vaccination in the presence of ATRA/NAFL significantly inhibited the binding of not only a homogeneous viral stain contained in the vaccine formulation, A/California/7/2009 H1N1, but also heterogeneous viral strains, A/New Caledonia/20/1999 H1N1 and A/Puerto Rico/8/1934 H1N1 (Figure 3E,F). These results further confirmed the synergistic adjuvant effect of ATRA and NAFL.

### The Mechanism Underlying Synergistic Adjuvant Effects of ATRA and NAFL

**FIGURE 4:**
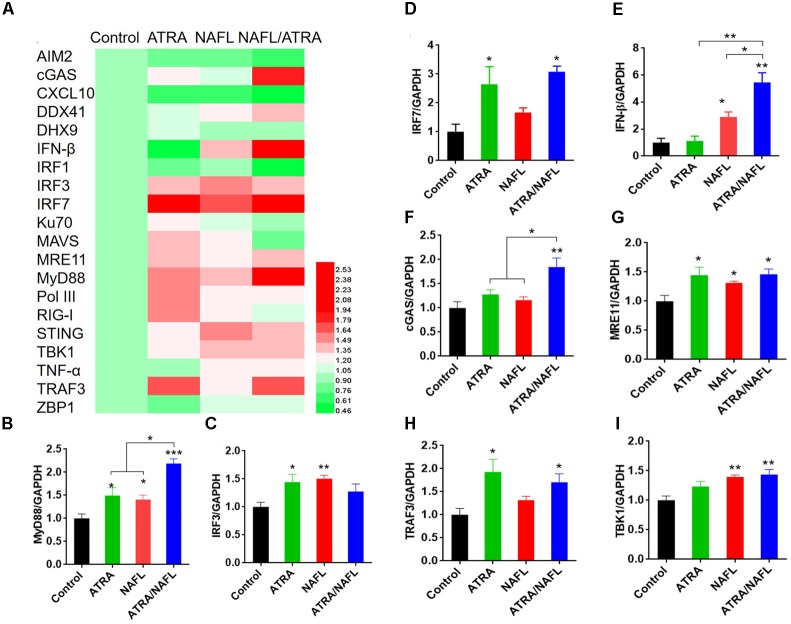
RNA expression level of skin at the inoculation site post-treatment of adjuvants. The lower dorsal back skin of C57BL/6 mice was exposed with ATRA, NAFL (high, 2 hits) and ATRA/NAFL for 24 h; then the skin was isolated, and RNA was extracted for qRT-PCR. **(A)** Heat map diagram of gene expression level of skin inoculation site. Changes in expression levels are displayed from green (downregulation) to red (upregulation), as shown in the color gradient at the bottom right corner. Rows indicate different genes and the columns represent samples post-treatment of ATRA, NAFL, ATRA/NAFL. Averages of replicates of different groups were shown. The heat map was illustrated using HemI 1.0 software. The mRNA expression level of **(B)** MyD88, **(C)** IRF3, **(D)** IRF7, **(E)** IFN-β, **(F)** cGAS, **(G)** MRE11, **(H)**, TRAF3 and **(I)** TBK1. *N* = 4. Data are presented as mean ± s.e.m. ^∗^*P* < 0.05; ^∗∗^*P* < 0.01 or ^∗∗∗^*P* < 0.001, respectively.

To investigate the underlying mechanisms of synergistic adjuvant effects of ATRA/NAFL, we determined the expression level of genes like dsDNA sensors and downstream genes at the skin inoculation site. The expression level of a cluster of genes was detected, including nucleic acid sensing signal system and cytokines possibly associated with ATRA and NAFL. The gene expression profile was summarized as a heat map and histograms (Figure 4). ATRA/NAFL synergistically upregulated two key adaptors for innate immunity signaling: MyD88 (Figures 4A,B) and IRF3/IRF7 (Figure 4A,C,D). However, a profound upregulation of IFN-β, but not TNF-α (Figures 4A,E), indicates that IRF3/IRF7 is the key pathway involved in the adjuvant effect of ATRA/NAFL. ATRA treatment alone did not contribute to the induction of IFN-β but upregulated a number of nucleic acid sensors, including cGAS, MRE11, Pol III, and DDX41 (Figure 4A,F,G). On the other hand, NAFL treatment contributed directly to the activation of IFN-β induction pathways (Figure 4A,C,D). As a result, the combination of NAFL and ATRA synergistically upregulated cGAS, MRE11, DDX41, STING, TRAF3, TBK1, IRF3, and IRF7, leading to a profound elevation of IFN-β (Figure 4A,C–I).

**FIGURE 5:**
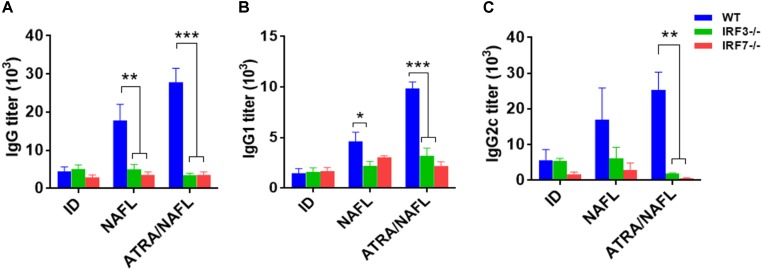
IRF3 and IRF7 play an important role in enhancing the humoral immunity of ATRA and NAFL. 5 μl SIV were immunized i.d. alone or with adjuvant ATRA or combined with NAFL (2 hits, high) in wild-type C57BL/6 mice, IRF3^-/-^ and IRF7^-/-^ knockout mice, and vaccine-specific antibody titer for **(A)** IgG, **(B)** IgG1 and **(C)** IgG2c were determined 2 weeks post-immunization. *N* = 4. Data are presented as mean ± s.e.m. ^∗^*P* < 0.05; ^∗∗^*P* < 0.01 or ^∗∗∗^*P* < 0.001, respectively.

To further confirm the crucial role of the nucleic acid sensing pathway in the adjuvant of ATRA/NAFL, IRF3 and IRF7 knockout mice were enrolled since they are common downstream adaptors in nearly all known type I interferon-producing nucleic acid sensing pathways. The SIV, including adjuvants, was immunized under the same conditions as those described above in wild-type C57BL/6 mice, IRF3^-/-^ and IRF7^-/-^ mice. Vaccine-specific antibody IgG (Figure 5A), IgG1 (Figure 5B) and IgG2c (Figure 5C) titers were determined 2 weeks post-immunization. In wild-type C57BL/6 mice, the NAFL group enhanced the titers of vaccine-specific antibodies IgG, IgG1 and IgG2c compared to the vaccine group, while the ATRA/NAFL group further elevated the titer of all antibodies in agreement with previous results. A significant decrease of total IgG generated by NAFL and NAFL/ATRA vaccination was noticed in both knockout mice (Figure 5A). Furthermore, the adjuvant effects of ATRA/NAFL on Th1 and Th2 immune responses were significantly reduced when the nucleic acid sensing pathway was not intact, as indicated by the inability of ATRA/NAFL to augment IgG1 and IgG2c titers (Figure 5B,C).

## Discussion

Influenza virus has a high mutation rate, and the emergence of a novel pandemic is unpredictable (Xu et al., 2013; Medina, 2018; Xie et al., 2018). Once a pandemic occurs, it will pose a great threat to human life and health. For example, in March 2009, a new swine-origin influenza, A (H1N1) pdm09, emerged in the United States, which then spread worldwide to 30 countries by human-to-human transmission in just 2 months, causing large numbers of deaths (Smith et al., 2009). During pandemics, it is common to see vaccine supplies dwindle, and developed countries receive vaccines much earlier than less developed countries (Kumar et al., 2012). We know that dose-sparing of influenza vaccine can be achieved by ID injection, calling for an adjuvant able to work for a pandemic vaccine via ID immunization (Kenney et al., 2004; Hung et al., 2012). In this study, we found that the topical application of ATRA primarily enhanced cutaneous influenza vaccination, especially via a Th2-biased immune response compared to the predominantly Th1 immune response initiated by NAFL. The combination of ATRA and NAFL synergistically balanced the Th1/Th2 immune responses and significantly strengthened the efficacy of both pandemic and SIVs.

Safety is the most important factor in vaccine development, and it cannot be ignored in the development of adjuvants, especially for intradermal immunization with higher adjuvant safety requirements. ATRA cream has been used clinically for many years, and NAFL has also been approved by the FDA for commercial use; thus, the safety of each has been widely recognized. Vitamin A has a reparative effect on skin wound caused by light damage. Therefore, it can provide a layer of bio-natural protective film for rapid protection of the laser-induced sterile inflammation microenvironment. Accordingly, the ATRA/NAFL combination does not introduce foreign chemicals to the body or cause damage to the outer surface of the skin. Furthermore, ATRA/NAFL activates the immune system without introducing any non-self molecules into the skin, and as such, it could be considered as an “all-natural” vaccine adjuvant, warranting a high level of safety.

**FIGURE 6:**
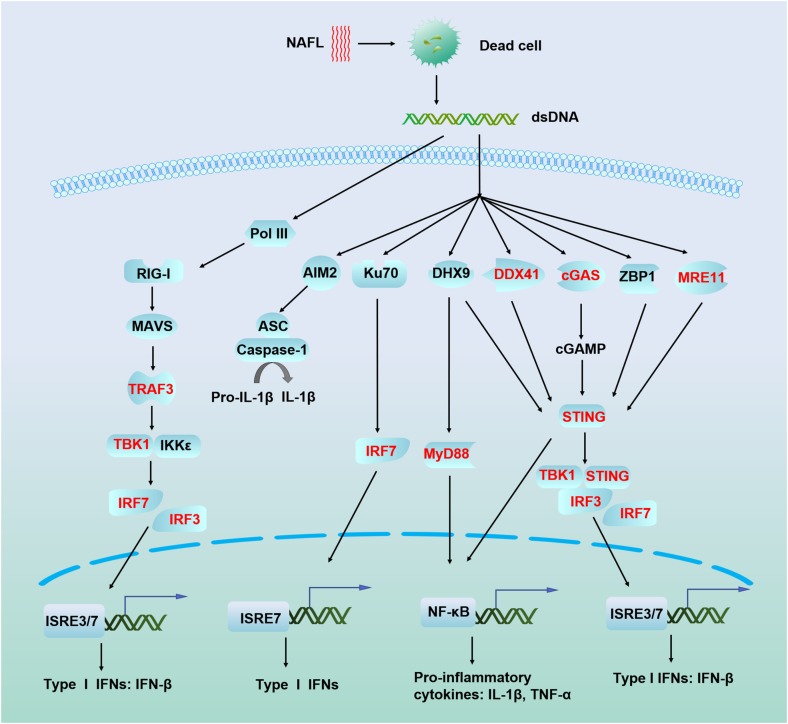
Schematic illustration of nucleic acid recognition and interferon production mechanism of ATRA/NAFL. Dead cells generated by NAFL or ATRA/NAFL released nucleic acid-like dsDNA in the cytoplasm which is sensed by many sensors leading to the production of type I IFNs via adaptors MAVS, STING, and transcriptional factors IRF3 and IRF7. Most DNA sensors like DHX9, DDX41, cGAS, ZBP1, and MRE11 are believed to activate STING, recruit TBK1 to phosphorylate IRF3/IRF7, and then dimerize and enter the nucleus, resulting in the production of type I IFNs. Pol III transcribes dsDNA to synthesize RNA, which is sensed by RIG-I, while RIG-I can be induced and activated by ATRA. RIG-I recruits TRAF3 via MAVS to trigger the activation of the downstream kinases TBK1 and IKK𝜀 and then phosphorylates IRF3 and IRF7, which homodimerize and enter the nucleus to induce the production of type I IFNs. Genes upregulated by ATRA/NAFL are shown in the bold red font. Abbreviations: AIM2, absent in melanoma 2; ASC, PYD and CARD domain containing (PYCARD); cGAMP, 2′3′ guanosine–adenosine monophosphate; cGAS, cyclic GMP–AMP synthase; DDX41, DEAD-box helicase 41; DHX9, DExH-box helicase 9; IFN-β, interferon beta; IKK𝜀, IkB kinase epsilon; IL-1β, interleukin 1 beta; IRF3, interferon regulatory factor 3; IRF7, interferon regulatory factor 7; ISRE, interferon-sensitive response element; Ku70, Lupus Ku autoantigen protein p70; MAVS, mitochondrial antiviral-signaling protein; MRE11, meiotic recombination 11 homolog A; MyD88, myeloid differentiation primary response 88; NF-κB, nuclear factor kappa light chain enhancer of activated B cells; Pol III, RNA polymerase III; RIG-I, RA inducible gene-I; STING, stimulator of interferon genes; TBK1, TANK binding kinase 1; TNF-α, Tumor necrosis factor alpha; TRAF3, TNF receptor associated factor 3; ZBP1, Z-DNA binding protein 1.

Non-ablative fractional laser has been previously demonstrated to enhance vaccination efficacy by inducing cell death and damage-associated molecular patterns (DAMPs), as noted above, particularly dsDNA (Wang et al., 2014, 2015). In the current study, we found that ATRA treatment synergistically strengthened dsDNA sensing and promoted the production of type I interferon in the skin. DAMPs are components of host cells released during cell damage or death. DAMPs initiate innate immune responses in the absence of infection, also called sterile inflammation. Sensing of cytosolic nucleic acids is a major mechanism by which the innate immune system detects foreign pathogens and cellular damage (Cai et al., 2014; Vance, 2016). To date, many cytosolic receptors of nucleic acids have been identified, and these are depicted in Figure 6. Among these receptors, cyclic GMP-AMP (cGAMP) synthase (cGAS) is a major cytosolic dsDNA sensor which triggers innate immune responses via the synthesis of the second messenger cGAMP. Subsequently, cGAMP binds to STING and recruits the downstream IFN-inducing proteins TBK1 and IRF3 (Burdette and Vance, 2013; Tsuchiya et al., 2016). Initial IFN-β signaling could trigger an autocrine loop that upregulates IRF7 and produces more type I interferons. Indeed, the adjuvant effect of NAFL depends on STING, IRF3, and IRF7, as further confirmed by the current study. ATRA is well known to induce the expression of RIG-I (Liu et al., 2000) which is responsible for dsRNA sensing involving TANK binding kinase-1 (TBK1) and IκB kinase epsilon (IKK𝜀) to promote the expression of type I and type III interferons through phosphorylation of IRF3 and IRF7 (Belgnaoui et al., 2011). Our study revealed that ATRA treatment not only induce RIG-I, which is in accordance with the previous studies (Balmer and Blomhoff, 2002) but also induce other nucleic acid sensors, especially cGAS, which upregulates the expression of STING, TBK1, TRAF3, IRF3, and IRF7, resulting in elevated IFN-β expression (Figure 6). Based on these results, we proposed a working mechanism of ATRA/NAFL such that NAFL provides the initial source, or dsDNA, to trigger nucleic acid sensing pathways, and ATRA treatment, or the combination, increases the number of key sensors and downstream factors to support a vigorous activation and production of type I interferons. ATRA/NAFL stimulates the induction and activation of pathways associated with RIG-I and STING, as discussed in many other reports in the literature, confirming that these two pathways are, to a certain extent, interrelated and may play an important role in immunity (Fischer et al., 2017; Zevini et al., 2017).

Our immunization experiments with IRF3 and IRF7 knockout mice also supported the conclusion that ATRA/NAFL’s adjuvanticity depends on the generation of type I interferon. Type I interferons can serve as molecular adjuvants by driving the maturation and migration of antigen presenting cells and supporting T- and B-cell survival, activation and differentiation (Tailor et al., 2006; Xu et al., 2014). Given the clear mode of action, our study raises the possibility that ATRA/NAFL could serve as a safe and effective adjuvant for skin vaccination for different types of influenza vaccines, including pandemic and SIVs. These studies shed light on the contribution of ATRA in strengthening the nucleic acid sensing pathway, inspiring the combinatorial use of ATRA with other immune stimulatory molecules which activate this pathway. As a micro-sterile infiammation array-based adjuvant, ATRA/NAFL merits further studies as a cutaneous adjuvant for various vaccines.

## Author Contributions

MW, LL, and SJ conceived and designed the experiments. PL, JW, and MC performed the experiments. PL, JW, QD, LL, MW, and SJ analyzed the data and wrote the manuscript.

## Conflict of Interest Statement

The authors declare that the research was conducted in the absence of any commercial or financial relationships that could be construed as a potential conflict of interest.

## Footnotes

1 http://www.who.int/mediacentre/factsheets/fs211/en/

2 https://www.accessdata.fda.gov/scripts/cder/daf/index.cfm?event=overview.process&applno=075264
